# From Atherosclerotic Plaque to Myocardial Infarction—The Leading Cause of Coronary Artery Occlusion

**DOI:** 10.3390/ijms25137295

**Published:** 2024-07-02

**Authors:** Ewelina Młynarska, Witold Czarnik, Piotr Fularski, Joanna Hajdys, Gabriela Majchrowicz, Magdalena Stabrawa, Jacek Rysz, Beata Franczyk

**Affiliations:** 1 Department of Nephrocardiology, Medical University of Lodz, ul. Zeromskiego 113, 90-549 Lodz, Polandgabrysia.majchrowicz@gmail.com (G.M.);; 2 Department of Nephrology, Hypertension and Family Medicine, Medical University of Lodz, ul. Zeromskiego 113, 90-549 Lodz, Poland

**Keywords:** myocardial infarction, atherosclerosis, cardiovascular disease

## Abstract

Cardiovascular disease (CVD) constitutes the most common cause of death worldwide. In Europe alone, approximately 4 million people die annually due to CVD. The leading component of CVD leading to mortality is myocardial infarction (MI). MI is classified into several types. Type 1 is associated with atherosclerosis, type 2 results from inadequate oxygen supply to cardiomyocytes, type 3 is defined as sudden cardiac death, while types 4 and 5 are associated with procedures such as percutaneous coronary intervention and coronary artery bypass grafting, respectively. Of particular note is type 1, which is also the most frequently occurring form of MI. Factors predisposing to its occurrence include, among others, high levels of low-density lipoprotein cholesterol (LDL-C) in the blood, cigarette smoking, chronic kidney disease (CKD), diabetes mellitus (DM), hypertension, and familial hypercholesterolaemia (FH). The primary objective of this review is to elucidate the issues with regard to type 1 MI. Our paper delves into, amidst other aspects, its pathogenesis, risk assessment, diagnosis, pharmacotherapy, and interventional treatment options in both acute and long-term conditions.

## 1. Introduction

The leading cause of mortality worldwide remains cardiovascular disease (CVD) [1]. It is estimated that over 4 million deaths annually in Europe and over 17 million deaths globally are attributable to CVD [2]. One of the most serious conditions associated with CVD is undoubtedly myocardial infarction (MI). It constitutes the most common cause of mortality in humans overall [3]. There are several types of MI that can be distinguished. Type 1 is associated with atherosclerotic plaque, specifically its erosion or rupture, leading to thrombus formation and subsequent vessel occlusion. Type 2 occurs when the oxygen supply to cardiomyocytes is insufficient relative to their demand for this element. In some patients presenting with symptoms suggestive of MI, sudden cardiac death may occur, referred to as type 3 MI. Additionally, type 4 MI is associated with percutaneous coronary intervention (PCI), while type 5 is related to coronary artery bypass grafting (CABG) [4]. However, the most common cause of MI is atherosclerosis, which leads to the occurrence of type 1 MI. Atheromatous plaque formation begins at a young age and progresses towards more unstable forms in parallel with lipid accumulation in the vessel wall and the ongoing inflammatory processes [5]. In our review, we are particularly interested in this type of MI.

## 2. The Pathogenesis of Atherosclerosis

The arterial vessel wall, progressing inward, comprises a singular layer of endothelial cells (ECs) forming the luminal boundary of the bloodstream. Beneath lies a layer predominantly composed of glycosaminoglycans and collagen, referred to as the “intima.” The subsequent layer of the vessel consists of smooth muscle cells (SMCs), also known as the “media,” while externally, it is encased by a fibrous layer termed the “adventitia” [6].

**Figure 1:**
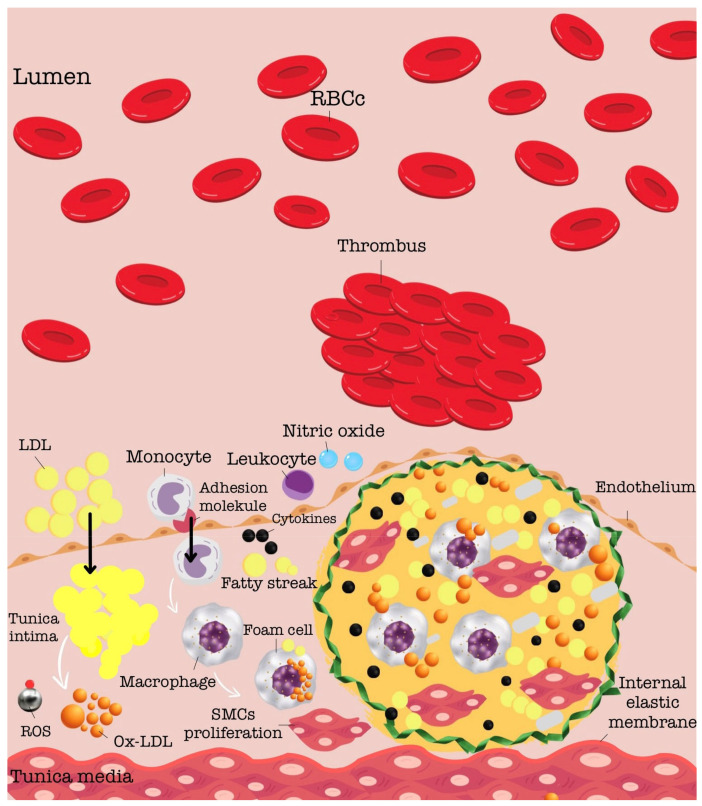
Figure depicting the pathogenesis of atherosclerotic plaque development [7]. Abbreviations: oxLDL—oxidised low-density lipoprotein, ROS—reactive oxygen species, RBC—red blood cells.

The development of atherosclerosis can be divided into three distinct stages: (a) the initial lipid-streak stage, (b) the fibrous plaque stage, and (c) the stage of advanced lesions and thrombosis [7] During the lipid-streak stage, various forms of lipids are retained and accumulate within the arterial walls. Macrophages infiltrate the intima and absorb excessive lipids, forming foam cells. In the subsequent phase, vascular smooth muscle cells (VSMCs) migrate to the innermost layer of the artery and form a fibrous cap over the atherosclerotic area [8]. This thick fibrous cap contributes to the stability of the plaque. However, excessive foam cell accumulation can lead to necrosis within the atherosclerotic plaques. As the disease progresses to the advanced lesions and thrombosis stage, the necrotic core expands while the fibrous cap weakens [9]. When damage occurs to the atherosclerotic plaque, the content of the necrotic core, upon direct contact with blood, interacts with coagulation factors and cells present in the blood, initiating the thrombosis process [10]. The pathogenesis of atherosclerotic plaque development is shown in Figure 1.

## 3. Risk Factors for Atherosclerosis

Atherosclerosis is one of the leading causes of serious organ-related complications that can lead to death or significant reduction in the quality of life for patients. Therefore, it is important to control risk factors that may contribute to the initiation or progression of the atherogenesis process. Known risk factors for atherosclerosis include dyslipidaemia, smoking, chronic kidney disease (CKD), obesity, hypertension, and diabetes mellitus (DM).

Numerous scientific studies have demonstrated that low-density lipoprotein cholesterol (LDL-C) is one of the primary risk factors for atherosclerosis. The concentration of LDL-C shows a positive correlation with the development of atherosclerosis [11,12]. LDL-C that has undergone modifications such as oxidation is taken up by scavenger receptors present in the arterial wall macrophages. The oxidation of LDL within the arterial walls leads to the production of inflammatory cytokines and the recruitment of inflammatory cells, ultimately exacerbating the atherogenic process [13].

Dyslipidaemia refers to clinical conditions characterised by abnormal concentrations of specific lipoproteins in the serum. These clinical states can be secondary to conditions such as diabetes, obesity, nephrotic syndrome, hypothyroidism, and cholestatic liver disease, or they may be genetically determined, leading to the development of familial hypercholesterolaemia (FH) [14]. FH is inherited in an autosomal dominant manner and is associated with elevated levels of LDL-C in the serum, which correlates with the occurrence of early complications related to atherosclerotic cardiovascular disease (ASCVD) as well as characteristic symptoms such as xanthelasmas, xanthomas, and corneal arcus. The occurrence of FH is primarily attributed to three genes: low-density lipoprotein receptor (LDLR)-binding defective variants in apolipoprotein B (APOB), loss-of-function mutations in the LDL-R gene, and gain-of-function mutations in the proprotein convertase subtilisin/kexin type 9 (PCSK9) gene [15,16]. FH can occur in a homozygous form, which is less common, but is associated with very high serum LDL-C levels and significantly worse prognosis compared to the heterozygous form. It can also occur in a heterozygous form, representing an intermediate phenotype between healthy individuals and those with homozygous FH [15].

Tobacco smoking is widely recognised as one of the most substantial risk factors for cardiovascular events. Smoking contributes to the initiation of atherogenesis through several key mechanisms, including endothelial dysfunction, reduction in high-density lipoprotein (HDL) levels, elevation of proatherogenic lipid levels and induction of inflammation [17].

CKD is a significant risk factor for the development of atherosclerosis. Processes contributing to the accelerated development of atherosclerosis in CKD patients include chronic inflammation, increased production of reactive oxygen species (ROS), impaired lipid and electrolyte metabolism, nitric oxide (NO) deficiency, mitochondrial damage, deoxyribonucleic acid (DNA) damage, and accumulation of uremic toxins [18]. Additionally, CKD is a significant factor in generating secondary, difficult-to-treat hypertension, which is another important factor in atherosclerosis [19].

Another significant risk factor for atherosclerosis is obesity. White adipose tissue (WAT) is one of the largest organs in the human body, accounting for 9–28% of body weight in lean adults to 40–70% in obese individuals [20]. It plays a significant role in mediating inflammatory processes due to the production of adipokines, which indirectly influence the development of atherosclerosis, and because of its macrophage content, which significantly varies depending on individual body weight. While macrophages in lean individuals primarily exhibit an anti-inflammatory phenotype, in obese individuals, macrophage activity tends to promote inflammation [21]. Adipokines produced by adipose tissue can be categorised into those that have protective effects on atherosclerosis, those that worsen atherosclerosis, and those with diverse functions. In obese individuals, the balance of adipokines is disrupted, leading to increased activity of adipokines that promote atherosclerosis processes. Adipokines regulate the inflammatory response and lipoprotein concentration, and indirectly influence the generation of foam cells [22].

DM is a significant factor exacerbating atherosclerosis, a correlation well substantiated in numerous scientific studies. One of the linking factors between diabetes and atherosclerosis is vascular calcification (VC), an irreversible condition associated with the deposition of calcium in arterial vessels. This results in loss of vascular elasticity, thickening of vessel walls, and increased arterial stiffness, leading to higher pulse wave velocity and intensifying the generation of subsequent atherosclerotic plaques [23,24]. Furthermore, oxidative stress induced by chronic hyperglycaemia is a primary pathogenetic mechanism of diabetes complications. This is because it modulates the inflammatory process, damages tissues, stimulates fibrotic processes, and affects the function of the vascular endothelium [25]. Patients with diabetes have elevated levels of inflammatory mediators such as C-reactive protein (CRP), plasminogen activator inhibitor 1 (PAI-1), and interleukin-6 (IL-6), which constitute another link between diabetes and atherosclerosis [26].

Hypertension is one of the most common chronic diseases worldwide, and is closely associated with atherosclerosis. The effector involved in the pathogenesis of hypertension, the renin–angiotensin–aldosterone system (RAAS), particularly angiotensin II, can stimulate inflammatory pathways through the master transcriptional regulator nuclear factor kB (NF-kB) [27]. Additionally, chronic overactivation of the RAAS leads to sustained stimulation of the sympathetic nervous system, directly affecting blood vessels by causing vasoconstriction and potentially leading to vascular fibrosis, which promotes the progression of atherogenesis. Moreover, the RAAS is closely linked with nicotinamide adenine dinucleotide phosphate hydrogen (NADPH) oxidase, an enzyme responsible for generating (ROS) [28].

## 4. Consequences of Atherosclerosis

Despite significant improvement in the prevention, diagnosis, and treatment of atherosclerosis, it remains the foremost contributor to global mortality, accounting for 18.6 million deaths in 2019 [29]. Atherosclerosis initiates its development at a young age, then progresses through a prolonged latent and asymptomatic stage characterised by the gradual accumulation of lipids within the atherosclerotic plaque and lipid-engorged cells. Subsequently, it becomes clinically significant in the late stage, when the atherosclerotic plaque significantly impedes blood flow through the vessels, resulting in a wide spectrum of symptoms [30]. Chronic impairment of blood flow through arterial vessels can lead to various conditions such as chronic coronary syndromes, stenosis of carotid and vertebral arteries, and narrowing of arteries in the lower and upper extremities [31].

The vulnerability to rupture largely depends on plaque type. The stability of the atherosclerotic plaque depends on the degree of cap inflammation and the thickness of the fibrous cap. The probability of rupture increases with a decrease in cap thickness, which is promoted by the breakdown of the extracellular matrix (ECM) and collagen, as well as the death of VSMCs. However, plaque rupture often occurs subclinically, as VSMCs have the ability to repair ruptures and reorganize associated thrombi. Complex plaques often exhibit indications of repeated ruptures and subsequent repair processes, ultimately leading to constriction of the vessel lumen [32]. With the progression of the atherosclerotic plaque, its most severe complication may occur—rupture and initiation of the thrombosis process. The process of atherosclerotic plaque rupture begins with the disruption of the innermost layer of the arterial vessel, the endothelial surface, leading to platelet adhesion facilitated by the binding of platelet receptors to collagen and von Willebrand factor within the matrix of damaged endothelial cells. Initiation of the coagulation process by active (a) factors VIIa, XIIa, and tissue factor leads to thrombin activation. Thrombin in turn acts via protease-activated receptors (PARs), triggering the release of thromboxane and adenosine diphosphate (ADP). These mediators act in a paracrine and autocrine manner, intensifying platelet activation and ultimately culminating in the formation of a massive clot. [5].

The occlusion of the arterial vessel lumen leads to ischaemia and consequently to severe dysfunction of the organ supplied by the respective artery. The most serious acute complications of atherosclerosis include acute ischaemia of internal organs and extremities, ischemic stroke, and MI [30]. MI due to atherosclerosis is a leading cause of mortality and disability worldwide and one of the most serious complications of atherosclerosis, which we plan to address with particular attention in our research endeavours.

## 5. Cardiovascular Disease Risk Assessment

CVD risk screening can be either opportunistic, meaning without a predefined strategy, or systematic, performed as a part of a formal assessment programme [33]. Systematic screening improves risk factors, but studies have shown that it does not benefit cardiovascular outcomes [34,35,36]. Meanwhile, opportunistic screening for ASCVD risk factors is recommended, as it allows for better detection, yet whether it is beneficial on clinical outcomes is not yet certain [37].

The main risk factors of CVD include age, sex, high blood pressure, cigarette smoking, DM, blood apolipoprotein B-containing lipoproteins, adiposity, and many more [33].

The European Society of Cardiology (ESC) provides recommendations for CVD risk assessment, which are shown in Table 1 below.

Individuals can be categorised based on their cardiovascular disease risk based on their current classification provided by the European Society of Cardiology shown in Table 2 below.

### 5.1. ASCVD Risk Estimation in Apparently Healthy People

Apparently healthy people are individuals that have not been diagnosed with ASCVD, DM, or any other severe disease [33]. As of now, current ESC guidelines from 2021 recommend using the updated version of the Systemic Coronary Risk Estimation (SCORE) algorithm—SCORE2—to estimate the risk of developing ASCVD in apparently healthy people aged 40 to 69 years [33]. SCORE2 provides several advantages over the previous SCORE model, as it was designed with larger derivation and validation cohorts, has been systematically recalibrated, accounts for the impact of competing risks by non-CVD deaths, and is able to estimate the risk non-fatal CVD, whereas SCORE estimated CVD mortality only [38,39]. The algorithm of SCORE2 includes risk factors such as age, sex, cigarette smoking, systolic blood pressure value, and the value of non-high-density lipoprotein cholesterol [38]. Thus, it is able to estimate the risk of fatal and non-fatal CVD (such as MI or stroke) events over a period of 10 years [38].

Most of the usual CVD risk prediction models tend to perform poorly in the elderly population, as has been observed in several studies [40,41]. That is usually due to their algorithms not taking into account the competing risk of non-CVD mortality, which leads to an overestimation of the actual 10-year risk of CVD and the treatment benefit [42]. Consequently, the ESC guidelines from 2021 recommend using the SCORE2—Older Persons (SCORE2-OP) algorithm to assess the ASCVD risk in apparently healthy people over 70 years old [33]. SCORE2-OP is a competing risk-adjusted model able to estimate the 5- and 10-year risk of incident CVD [43]. It can also be used to calculate the absolute CVD risk reduction achieved by reaching individual treatment goals [43]. Like the SCORE model, it includes the same risk factors and assesses the combined risk of both fatal and non-fatal CVD events [38,43].

Both the SCORE2 and the SCORE2-OP were designed using the same data sources and risk regions, and therefore individuals can progress from using the SCORE2 model to the SCORE2-OP as they naturally get older [38,43]. SCORE2 and SCORE2-OP categorize patients into different CVD risk groups according to their age, as shown in Table 3 below. These categories translate to different treatment recommendations: in the low- to moderate-CVD-risk category risk factor, treatment is generally not recommended, in the high-CVD-risk category risk factor, treatment should be considered, and in the very high-CVD-risk category risk factor, treatment is generally recommended [33]. Additionally, the treatment recommendation for lipid-lowering drugs in apparently healthy people over the age of 70 may be considered, as it is a Class IIb recommendation [33].

Noteworthily, SCORE2 and SCORE2-OP are calibrated to four categories of mostly European countries that are grouped into the categories of low, moderate, high, and very high CVD risk based on national CVD mortality rates published by the World Health Organization (WHO) [33]. However, in different regions of the world, different risk estimation models are used, for example, the Framingham risk score [44].

### 5.2. ASCVD Risk Estimation in Patients with Established Atherosclerotic Cardiovascular Disease

Patients with an established ASCVD are usually at a very high risk of recurrent CVD events if risk factors are not treated [33]. This population require different risk stratification tools to estimate their recurrent CVD risk. The commonly used models include the Secondary Manifestations of Arterial Disease 2 (SMART2) risk score and the European Action on Secondary and Primary Prevention by Intervention to Reduce Events (EUROASPIRE) risk model [33,45].

The SMART2 risk model is an updated version of the previous SMART risk model that is used to estimate 10-year residual risk of fatal and non-fatal ASCVD event risk in patients with established ASCVD (understood as coronary artery disease, cerebrovascular disease, or peripheral artery disease) aged 40 to 80 years [45]. SCORE2 performs better than other existing tools, such as the previous SMART-REACH or the EUROASPIRE risk calculator, because of several new features [45,46,47]. For instance, it is widely geographically recalibrated to multiple different risk regions, which improves its clinical utility [45]. Moreover, the model accounts for the impact of competing risks, which neither the original SMART score nor the EUROASPIRE risk calculator does, which prevents the overestimation of predicted risk in the elderly [45,48].

The EUROASPIRE risk model is a secondary prevention tool able to estimate 2-year risk of recurrent major and non-major cardiovascular events in patients aged <75 years with stable coronary artery disease [47]. According to De Bacquer et al. [47] the EUROASPIRE model outperformed the previous SMART model for SMART-CAD (SMART-coronary artery disease) patients in predicting outcomes such as CVD death, non-fatal MI, or stroke.

### 5.3. ASCVD Risk Estimation in Persons with Type 2 Diabetes Mellitus

Most patients diagnosed with DM2 are considered to be at high ASCVD risk, while patients with additional severe target organ damage (TOD) are at very high CVD risk; however, patients with well-controlled short-standing DM, no evidence of TOD, and no additional ASCVD risk factors may be considered to be at moderate CVD risk [49].

There are specific risk models that estimate the CVD risk in patients with type 2 DM [33]. These algorithms usually include factors such as: duration of DM, glycated haemoglobin (HbA1c) level, and presence of TOD [33]. For instance, there are the ADVANCE (Action in Diabetes and Vascular Disease: Preterax and Diamicron-MR Controlled Evaluation) risk score and the UKPDS (UK Prospective Diabetes Study) risk engine [33]. The ADVANCE risk score predicts 10-year CVD risk, while the UKPDS predicts fatal and non-fatal CVD risk [50,51]. However, the ESC guidelines from 2021 do not recommend the use of these calculators [33].

### 5.4. Lifetime ASCVD Risk Estimation

There are risk-calculating algorithms that are able to estimate lifelong CVD risk, an example being the LIFE-CVD (Lifetime-Perspective Cardiovascular Disease) calculator [52]. This model takes into account the predicted risk in the context of competing risks from other diseases over the remaining expected lifespan of an individual and is able to identify high-risk patients in the short and medium term [52]. Moreover, it is able to estimate the lifetime benefit of preventive interventions, which may be helpful in clinical practice in communicating with an individual patient [52].

## 6. Myocardial Infarction

### 6.1. Pathogenesis

Type 1 MI is closely related to atherosclerotic and thrombotic coronary artery disease [4,53]. This term is used to describe a heart attack that is caused by a destabilised atherosclerotic plaque that has been damaged (rupture, erosion) [54,55,56]. Studies show that the vast majority (73%) of coronary artery thrombosis is caused by a ruptured atherosclerotic plaque [57]. We define a ruptured atherosclerotic plaque as a gap in the “fibrous cap” of the atherosclerotic plaque that separates the necrotic core from the lumen of the coronary artery [57,58]. Another important mechanism that may lead to the development of acute thrombosis is the erosion of an atherosclerotic plaque. In the case of erosion of the atherosclerotic plaque, endothelial cells are lost and the intima, consisting mainly of SMCs and extracellular matrix, is exposed [57,59]. As a result of these changes, a thrombus is formed, which leads to coronary vessel occlusion, which in turn results in reduced blood flow to the heart, ischaemia of cardiomyocytes, and ultimately necrosis [53,55].

### 6.2. Symptoms

A very important aspect of MI is early recognition of symptoms, which allows for quicker access to medical help and also influences the morbidity and mortality of patients [60]. The most typical symptom of a heart attack is chest pain radiating from the left shoulder to the neck, shortness of breath, feeling of anxiety, profuse sweating, nausea, vomiting, or a combination of the described symptoms [56,61]. The pain is typically sudden and intense, characterised by a sensation of pressure and lasting more than 10 min. In addition to the aforementioned symptoms, the patient may also report dizziness and a feel of approaching death [56]. We also distinguish the so-called “silent” heart attack, not accompanied by any specific symptoms despite an insufficient amount of blood supplying the heart muscle [56,61,62].

### 6.3. Diagnostic Tools

#### 6.3.1. Electrocardiogram

When a patient presents with the above symptoms, the basic investigation is a 12-lead electrocardiogram (ECG). This can be used to differentiate between patients with permanent ST-segment elevation MI (STEMI) and patients without permanent ST-segment elevation, which means non-ST-elevation acute coronary syndrome (NSTE-ACS) [63].

Patients with STEMI are those who have new ST-segment elevation in at least two contiguous leads on the ECG [63]. Elevation includes ≥2.5 mm in men <40 years, ≥2 mm in men >40 years or ≥1.5 mm in women regardless of age in leads V2 and V3 and/or ≥1 mm in the other leads [63]. These criteria include all patients except those with left ventricular hypertrophy and those with left bundle branch block (LBBB) [63]. It is important to obtain additional right ventricular leads (V3R and V4R leads) when STEMI of the inferior wall is suspected to assess for possible ST elevation [64]. In addition, posterior leads (V7–V9) may be recorded if the patient is symptomatic and the ECG is equivocal [63]. In either case, an ST-segment elevation ≥0.5 mm is sufficient for diagnosis.

Patients with NSTE-ACS can present with a variety of ECG variants, including non-permanent ST elevation, persistent or transient ST depression, and abnormal T waves, including T wave inversion, hyperacute T wave, or biphasic T wave [65,66,67,68,69]. In more than a third of cases, the ECG may be normal [63].

#### 6.3.2. Biomarkers

**Figure 2:**
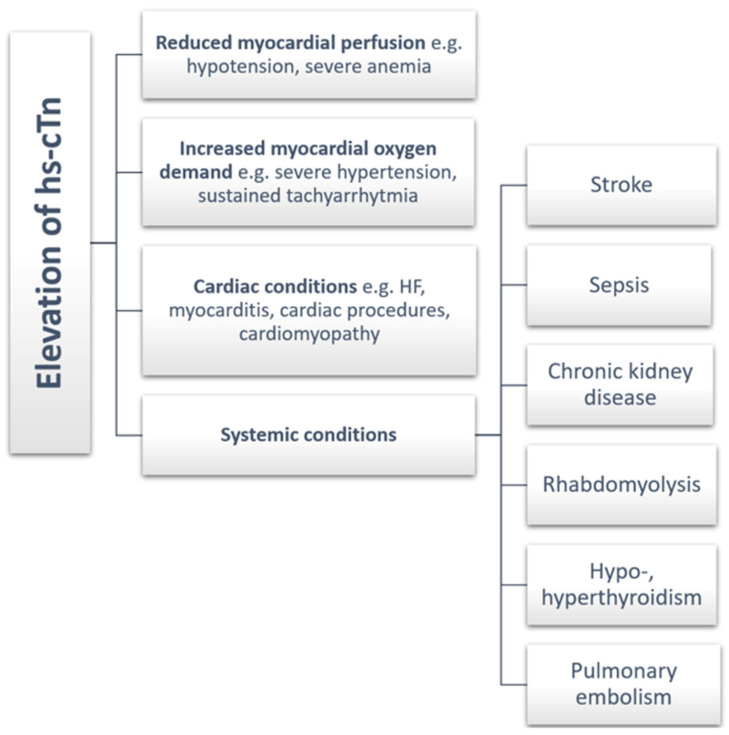
Conditions that may increase hs-cTn levels [1,11]. Abbreviations: hs-cTn, high-sensitivity cardiac troponin; HF, heart failure.

In all patients with suspected ACS, it is important to obtain high-sensitivity cardiac troponin (hs-cTn) levels as soon as possible to assess the extent of myocardial damage [63,70,71,72]. They also have a complementary role in the diagnosis, risk assessment and subsequent management of patients. A rise above the 99th percentile upper reference limit allows the diagnosis of MI according to the criteria of the fourth universal definition of MI [4]. In patients with MI, troponin levels rise very rapidly from the onset of symptoms and then persist for up to several days [4,70,73,74]. However, it is important to remember that troponins increase with myocardial damage, not only in MI. Conditions in which they may also be elevated are shown in Figure 2.

In addition, it is also important to consider that clinical variations such as age or sex may also be relevant to baseline troponin levels [75,76,77]. Nevertheless, absolute changes in hs-cTn levels have diagnostic and prognostic value [63].

It is recommended that a second measurement of hs-cTn levels should be performed according to the 0 h/1 h or 0 h/2 h algorithm to shorten the time to diagnosis. On this basis, patients are classified into a rule-out pathway, a rule-in pathway, or an observation pathway [63,73,74,78,79,80]. In the rule-out pathway, patients present with very low baseline hs-cTn levels or low baseline hs-cTn levels and no growth after 1 h/2 h. Patients with high baseline hs-cTn levels or growth after 1 h/2 h are eligible for the rule-in pathway. In contrast, patients who do not meet the criteria for either pathway remain on the observation pathway. Studies show that mortality in this group of patients is similar to that of patients in the rule-in pathway [81]; therefore, these patients will have an additional hs-cTn measurement at 3 h and echocardiography may be performed to determine further management [82,83].

#### 6.3.3. Non-Invasive Imaging

##### Echocardiography

If acute coronary syndrome (ACS) is suspected and the diagnosis is uncertain, transthoracic echocardiography may provide evidence of changes consistent with ongoing ischaemia or previous MI [4,63]. In addition, echocardiography should be performed to assess left and right ventricular function or to investigate possible mechanical complications of the infarction if the patient is haemodynamically unstable or in cardiogenic shock [63]. However, if acute coronary artery occlusion is suspected, echocardiography should not delay transport of the patient to the haemodynamic laboratory [63].

##### Computed Tomography

In patients with suspected ongoing ACS, particularly those with suspected coronary artery occlusion, computed tomography is not indicated or recommended [63]. In patients on the observation pathway in whom the hs-cTn and ECG remain equivocal, angiographic computed tomography of the coronary arteries may be performed [63]. A normal scan, ruling out the presence of atherosclerotic plaque, is of high diagnostic value and allows the exclusion of ACS [63]. In patients who are in the rule-out pathway, performing computed coronary tomography angiography (CCTA) may allow detection of the presence of atherosclerotic plaque and thus indicate prophylactic treatment in these patients [84]. It is also the preferred diagnostic test for ruling out the differential diagnosis of potentially life-threatening ACS such as pulmonary embolism (PE) or aortic dissection [63]. However, CCTA is of limited use in patients with tachycardia, stents, known coronary artery disease (CAD), or extensive coronary calcification.

##### Magnetic Resonance

Cardiac magnetic resonance is preferred when a diagnostic assessment of the myocardium cannot be obtained by echocardiography [63]. The use of paramagnetic contrast agents allow assessment of myocardial perfusion and the extent of myocardial damage, providing information on myocardial viability and scarring [4,63]. It can be used to differentiate acute MI from, for example, myocarditis or Takotsubo cardiomyopathy in cases of diagnostic uncertainty [63]. In addition, it plays a special role in the diagnosis of patients with suspected MI with non-obstructed coronary arteries (MINOCAs) following invasive angiography and is the gold standard for the assessment of left ventricular thrombus [63].

### 6.4. Acute Pharmacotherapy

#### 6.4.1. Oxygen

Supplementation with oxygen is recommended for patients with acute coronary syndrome who are hypoxemic, defined as oxygen saturation < 90% [85].

#### 6.4.2. Nitrates

Sublingual nitrates may be used to alleviate ischaemic symptoms. However, caution should be exercised, as resolution of symptoms does not necessarily mean resolution of the ACS, and a 12-lead ECG should be obtained [63,86]. Nitrates should be avoided in patients with hypotension, significant bradycardia or tachycardia, right ventricular infarction, and known severe aortic stenosis or use of phosphodiesterase-5 (PDE-5) inhibitors in the preceding 24–48 h [63].

#### 6.4.3. Pain Relief

The patient may be given intravenous morphine to reduce severe pain. It has been observed that morphine can reduce myocardial and microvascular damage in patients with persisting acute coronary occlusion [87], which may be related to a reduction in oxygen demand due to a reduction in preload and negative inotropic and chronotropic effects [63]. Although morphine may slow the absorption of oral drugs from the gastrointestinal tract, it may also delay the onset of action of antiplatelet drugs [88,89,90].

#### 6.4.4. Beta-Blockers

In experimental studies, metoprolol has shown the greatest cardioprotective effect of the beta-blockers and is currently the most commonly used in trials in patients undergoing primary PCI [91,92]. It has been shown to be associated with a reduction in the incidence of ventricular fibrillation and microvascular obstruction [91,92,93,94]. It can therefore be used safely in patients with STEMI who have no symptoms of acute heart failure and no other contraindications [91,92,93].

#### 6.4.5. Antithrombotic Therapy

One of the most important components of treatment for patients presenting with symptoms of ACS is anticoagulant therapy [63]. The type and duration of anticoagulant treatment varies from patient to patient, depending on individual predisposition and the type of procedure performed. It is important to balance the benefits of anticoagulant treatment with the risk of acute life-threatening bleeding [95,96].

##### Antiplatelet Therapy

Antiplatelet drugs have an important role in treatment in the acute phase of acute coronary syndrome. Based on trial results, dual-antiplatelet therapy (DAPT) consisting of aspirin and a P2Y12 receptor inhibitor is recommended for patients with ACS [97,98]. Among P2Y12 inhibitors, prasugrel is preferred to ticagrelor in patients undergoing PCI [63]. This followed trials showing that prasugrel was associated with a reduction in the composite endpoint of death, MI or stroke compared with ticagrelor, without an increase in haemorrhagic complications [99]. Clopidogrel is not preferred unless there are contraindications or unavailability of the aforementioned drugs or the patient is at high risk of bleeding [100]. It may also be considered in patients over 70 years of age [101]. Cangrelor is the only P2Y12 receptor inhibitor that is administered intravenously. Its use may be justified in patients with ACS undergoing PCI who cannot receive a P2Y12 inhibitor orally (due to cardiogenic shock or mechanical ventilation), as it has been shown to be effective in preventing intra-procedural and post-procedural stent thrombosis [63,102,103]. Glycoprotein (GP) IIb/IIIa inhibitors are not routinely used in patients with ACS undergoing coronary angiography. However, their use should be considered in cases of no-reflow or thrombotic events during PCI [63,104]. In addition, GP IIb/IIIa inhibitors should be considered in high-risk PCI patients who have not previously received a P2Y12 receptor inhibitor [104].

Aspirin in patients with ACS should be given at a loading dose as soon as possible and then switched to maintenance therapy [105,106]. In patients with STEMI undergoing PPCI, pre-treatment with a P2Y12 receptor inhibitor may be considered, but is not recommended in patients with NSTE-ACS if the coronary anatomy is unknown [99,107,108]. Patients undergoing PCI who have not received pretreatment with a P2Y12 receptor inhibitor receive a saturating dose at the time of invasive treatment [63].

Maintenance of antiplatelet therapy after revascularisation is discussed in the subsection on long-term treatment.

##### Anticoagulant Therapy

Treatment with anticoagulants is also an important part of the initial and perioperative management of patients with ACS who are being treated invasively. Consequently, anticoagulants are recommended for all patients at the time of diagnosis of ACS [109].

Anticoagulant treatment is continued until the end of invasive treatment and should then be discontinued [63]. Exceptions include patients with atrial fibrillation or left ventricular aneurysm with thrombus formation [63].

For STEMI patients undergoing PPCI, unfractionated heparin is recommended at the time of the procedure due to its favourable risk–benefit profile [63]. Alternatively, enoxaparin or bivalirudin can be used, as both drugs have shown beneficial effects in clinical trials, resulting in a reduction in primary endpoints, including death and major bleeding [110,111]. In addition, bivalirudin is recommended as an alternative in patients with a history of heparin-induced thrombocytopenia.

Anticoagulants are also recommended for patients with NSTE-ACS undergoing immediate or early (<24 h) invasive angiography and possibly PCI if required [63]. Unfractionated heparin is the drug of choice, although clinical trials have shown that the administration of enoxaparin does not result in a difference in mortality or major bleeding compared with unfractionated heparin [112]. Therefore, both drugs can be used interchangeably. If a patient with NSTE-ACS does not undergo early invasive angiography, pharmacotherapy alone with fondaparinux is used as initial treatment [113]. It is preferred to enoxaparin. However, a full bolus of unfractionated heparin is recommended at the time of invasive treatment because of the risk of thrombus formation in the catheter. If fondaparinux is not available, enoxaparin should be used [63].

### 6.5. Invasive Management Strategies

The choice of management strategy in the acute phase of ACS depends on the initial ECG assessment and the haemodynamic stability of the patient. Once the patient’s condition has stabilised and the symptoms of ACS have resolved, the decision to repeat PCI or CABG can be made later in the hospital course [63].

#### 6.5.1. PCI

According to ESC guidelines, patients with STEMI should undergo immediate (within 120 min of diagnosis) primary PCI (PPCI) [63,114,115]. In randomised clinical trials, PPCI with drug-eluting stent implantation has been shown to be superior to fibrinolysis, with significant reductions in mortality, reinfarction, and stroke [116,117]. However, if it is not possible to perform PPCI within 120 min, then fibrinolysis is recommended [63]. In addition, fibrinolysis is preferred as part of the pharmaco-invasive strategy for ACS if the patient presents within 12 h of symptom onset [63,118]. After successful fibrinolysis (i.e., improvement in ischaemic symptoms, >50% reduction of ST-segment elevation, haemodynamic stability achieved), the patient should undergo early invasive angiography [117,119]. On the other hand, if fibrinolysis has been ineffective, i.e., ST-segment elevation less than 50% within 60–90 min after fibrinolytic administration, electrical or haemodynamic instability, worsening ischaemia or persistent chest pain, the patient is a candidate for urgent PCI [120,121,122].

Patients with STEMI presenting more than 48 h after symptom onset and without persistent symptoms should not undergo routine PCI, as this offers no benefit over medical management alone, consistent with the management of patients with chronic coronary syndromes [63,123,124,125].

**Figure 3:**
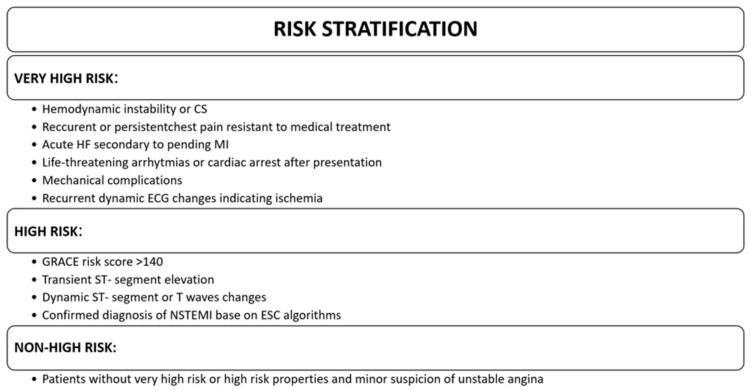
Risk criteria for patients with NSTE-ACS. Abbreviations: CS cardiogenic shock; HF heart failure; MI myocardial infarction; ECG electrocardiogram, NSTEMI non-ST-elevation myocardial infarction.

Patients with NSTE-ACS undergo immediate (very high risk), early (high risk), or elective (not high risk) invasive strategies, depending on the level of risk [63]. The risk criteria for patients with NSTE-ACS are shown in Figure 3.

In patients at very high risk of NSTE-ACS, the ESC guidelines recommend an immediate invasive strategy with urgent coronary angiography and PCI if necessary [63]. Patients at high risk of NSTE-ACS should be managed with an early (within 24 h) invasive strategy [63]. In other patients with suspected NSTE-ACS and non-elevated troponins or with elevated troponins but not meeting criteria for MI, the decision on strategy is based on suspicion. Patients diagnosed with NSTEMI or with high suspicion of unstable angina in patients with NSTE-ACS undergo a routine invasive strategy with coronary angiography [63]. Meta-analyses have shown that the use of a routine invasive strategy does not reduce the risk of all-cause mortality, but does reduce the risk of a composite ischaemic endpoint [126,127]. However, it is important to note that it may increase the risk of peri-procedural complications and bleeding. A selective invasive strategy is recommended for patients with low suspicion [63].

Patients with MI caused by multivessel disease are becoming more common, and therefore there is an increasing number of trials evaluating the effect of PCI of the infarct-related artery (IRA) only versus complete revascularization. Some of these trials have shown that complete revascularization reduces the risk of cardiovascular death or repeat MI more than PCI of the IRA [128,129,130].

#### 6.5.2. CABG

CABG should be considered if the STEMI patient has failed PCI, has extensive multivessel disease, or has symptoms of cardiogenic shock. It is a safe and feasible therapeutic option, although it is important that the IRA is not occluded [63,131]. CABG is also recommended for patients who need both revascularization and surgical repair of mechanical complications of a heart attack [63,132]. However, there is still a need for randomised control trials to establish the optimal time at which CABG should be performed [131].

### 6.6. Long-Term Treatment

#### 6.6.1. Cardiac Rehabilitation, Physical Activity, Smoking Cessation

After an ACS, patients should undergo secondary prevention to reduce the risk of recurrent MI [133]. The most effective way to do this is to participate in cardiac rehabilitation, which aims to provide patients with multidisciplinary care supervised and delivered by a team of doctors, physiotherapists, and dieticians [134]. Cardiac rehabilitation should begin as soon as possible after the onset of ACS and should include assessment of the patient’s health, control of cardiovascular risk factors, recommendation of physical exercise, dietary advice, encouragement to give up smoking, and psychological support [133].

#### 6.6.2. Pharmacotherapy

##### Antithrombotic Therapy

According to ESC guidelines, DAPT should be continued for 12 months after PCI unless contraindicated [63,135]. In some cases, DAPT may be shortened, extended, or modified.

In patients at high risk of bleeding on DAPT, the duration of DAPT may be shortened to 1, 3 or 6 months, or the patient may be de-escalated from prasugrel/ticagrelor to clopidogrel [63,136]. De-escalation should occur no earlier than 30 days after ACS [136]. Shortening DAPT in patients should be considered if no cardiovascular events have occurred after 3–6 months and patients are not at high risk of ischaemia [137,138,139]. If DAPT is shortened, P2Y12 inhibitor monotherapy is preferred to aspirin. In patients at high risk of bleeding, DAPT for one month and then switching to P2Y12 monotherapy or aspirin may be considered [63,140].

In contrast, prolonged DAPT should be considered in patients at high thrombotic risk and without an increased risk of major or life-threatening bleeding [141].

For patients who were on anticoagulants before the ACS, triple-anticoagulant therapy (TAT) is recommended after the ACS for up to one week or up to one month [63,142]. They are then switched to dual-anticoagulant therapy (DAT), consisting of an oral anticoagulant (OAC) and single-antiplatelet therapy (SAPT), preferably clopidogrel, for up to 12 months after the ACS [63,142]. DAT may be shortened to 6 months if the patient has multiple high-risk factors for bleeding [143].

##### Lipid-Lowering Therapy

In patients who have had an ACS, it is recommended that LDL-C levels be maintained below 55 mg/dL, which is associated with a lower risk of cardiovascular events [144]. For a repeat cardiovascular event within the last 2 years, an LDL-C level < 40 mg/dL is recommended [144].

Lipid-lowering treatment should be started after an ACS during hospitalisation [63,144]. According to the ESC guidelines, a high-intensity statin, such as atorvastatin or rosuvastatin, at the highest tolerated dose is recommended to achieve adequate LDL-C levels [144,145]. The dose should be increased in patients taking a moderate-intensity statin during hospitalisation before the onset of ACS [144]. Lipid levels should be assessed 4–6 weeks after a change in treatment or dose to check that the target has been achieved and that the patient is tolerating the medication well [144]. Ezetimibe should be added in patients who are on the maximum tolerated dose of a statin without achieving adequate LDL-C levels or in patients with very high LDL-C levels for whom statin therapy alone is not sufficient [146]. In the case of a patient whose goal is not achieved with statins and ezetimibe, the addition of a PCSK9 inhibitor to lipid-lowering therapy is recommended [147].

##### Beta Blockers

Currently, beta-blockers are recommended for patients with ACS and reduced left ventricular ejection fraction (LVEF), regardless of the presence of heart failure (HF) symptoms [148,149]. In other cases, the use of beta-blockers should be considered according to ESC guidelines [63]. However, it should be borne in mind that some of the trials are outdated, either because they predate reperfusion or because the results are inconclusive. Some trials report that the use of beta-blockers is beneficial regardless of LVEF, while others report the opposite. There is also a similar discrepancy regarding the timing of such therapy [149,150,151].

##### RAA Inhibitors

Angiotensin-converting enzyme inhibitors (ACEI), or angiotensin receptor–neprilysin inhibitors (ARNIs) interchangeably are recommended in patients with ACS and concomitant HF symptoms, LVEF less than 40%, DM, HT, and/or chronic renal failure [148]. Their use has been shown to significantly reduce mortality within 30 days of MI [152]. Mineralocorticoid receptor blockers may also be used if the above drugs are intolerable [153]. In other patients, ACEI may be considered [63].

##### Antidiabetic Medications

In patients with ACS and concomitant DM sodium–glucose cotransporter type 2 (SGLT2) receptor inhibitors or glucagon-like peptide 1 (GLP-1) receptor agonists are recommended for glucose-lowering therapy [154]. In addition to lowering blood glucose levels, they have been shown to reduce major adverse cardiovascular events and cardiovascular death [154].

##### Proton Pump Inhibitors

In patients treated with antiplatelet drugs, proton pump inhibitors (PPIs) reduce the risk of bleeding [155]. Importantly, they have been shown not to interact with these drugs [156,157]. They are also indicated in patients receiving anticoagulant treatment, which is associated with a high risk of gastrointestinal bleeding [63].

**Figure 4:**
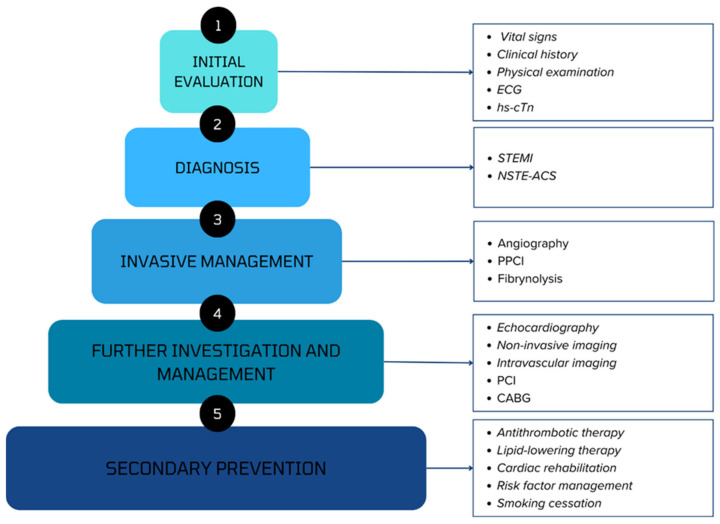
An overview of the comprehensive management of patients presenting with ACS. Abbreviations: ECG, electrocardiogram; hs-cTn, high-sensitivity cardiac troponin; STEMI, ST-elevation myocardial infarction; NSTE-ACS, non-ST-elevation acute coronary syndrome; PPCI, primary percutaneous coronary intervention; CABG, coronary artery bypass grafting.

An overview of the comprehensive management of patients presenting with ACS is provided in Figure 4.

### 6.7. Complications of MI

Cardiogenic shock (CS) is the main cause of in-hospital mortality in patients after acute MI [158,159,160]. Mortality in patients 30 days after MI is estimated at 40% and after 1 year at 50% [158]. The basic treatment regimen for CS includes immediate revascularization of the coronary artery, which improves the patient’s prognosis [158,161]. In addition to CS, HF is an important haemodynamic complication. Due to the increased risk of death, the problem should be diagnosed early and appropriate therapy initiated. Treatment methods with proven effectiveness include early reperfusion, ACEIs, and mineralocorticoid receptor antagonists (MRAs) [162]. Another complication of MI is cardiac arrhythmia [163]. In the period of reperfusion, the incidence of both ventricular and supraventricular arrhythmias decreased, and survival outcomes improved in patients with high-degree atrioventricular block as a complication of MI [163]. Another complication of acute MI with high mortality is left ventricular free-wall rupture (LVFWR). The incidence of this complication was estimated at 2% in patients after acute MI in the pre-thrombolytic era [164]. However, the latest studies show that in the era of reperfusion, the incidence of this complication has decreased and complicates 0.01–0.5% of patients with MI [165,166]. A very important consideration is quick diagnosis, which may enable effective surgery to repair the damaged wall [164,167]. It cannot be ignored that the operative mortality rate is high, amounting to 32% [165]. Patients who have had a first episode of acute MI may have another heart attack. We distinguish the following terms: recurrent MI and re-infarction. We use the term recurrent MI clinically to refer to an acute MI that occurred 28 days after the incident of MI, while we talk about re-infarction when the features of an acute MI occur within 28 days of the first or repeated infarction [4].

## 7. Prophylaxis of Atherosclerosis

Type 1 MI is fundamentally rooted in atherosclerotic changes, highlighting that effective prevention of atherosclerosis development can reduce the likelihood of future MIs. Interventions that slow the atherosclerotic process encompasses a broad spectrum of therapeutic actions, including increasing physical activity, smoking cessation, dietary modification, and the use of medications that protect cardiovascular health. It is widely known that obesity is a major contributing factor to atherosclerosis development, which can be effectively prevented and treated through dietary modification.

### 7.1. Physical Activity and Diet Changes

It is widely known that physical exercise leads to reduction in body mass, intensifying fat-burning processes, and thereby preventing obesity. During endurance exercise, intramuscular lipolysis and adipose tissue are regulated by hormonal mechanisms. The primary mechanism involves epinephrine acting through perilipin 1 activation and hormone-sensitive lipase (HSL). Additionally, during prolonged exercise, activity of the tricarboxylic acid cycle, the electron transport system, and β-oxidation enzymes increases, resulting in enhanced transport of fatty acids across mitochondrial membranes [168]. Furthermore, evidence suggests that elevated levels of physical activity and cardiorespiratory fitness (CRF) effectively mitigate the adverse effects of traditional cardiovascular risk factors, such as metabolic syndrome. Regular physical activity, even when initiated in old age, improves quality of life and contributes to slowing the progression of atherosclerosis [169,170].

The evidence consistently demonstrates that for healthy adults, a diet low in salt and animal-based foods combined with higher consumption of plant-based foods such as whole grains, vegetables, fruits, legumes, and nuts is associated with a reduced risk of acquiring atherosclerosis [171]. Weight loss has a significant impact on glucose metabolism, lipid profiles, and systemic inflammation. It can lead to decreases in blood pressure, fasting glucose levels, and haemoglobin A1c (HbA1c), as well as slow the progression of diseases [172].

### 7.2. Smoking Cessation

Tobacco smoking is recognised as a major risk factor for cardiovascular events.

The primary mechanisms by which smoking promotes atherogenesis include causing endothelial dysfunction, triggering inflammation, increasing proatherogenic lipid levels, and decreasing HDL levels [17]. Despite the well-known detrimental effects of smoking, many patients find it extremely challenging to quit, due to nicotine’s strong addictive properties. During the cessation process, individuals may encounter physical withdrawal symptoms such as difficulty concentrating, increased appetite, insomnia, restlessness, and anxiety [173]. Pharmacological treatments can help mitigate withdrawal symptoms and improve the chances of sustained abstinence. Recent evidence highlights the effectiveness of medications like varenicline, cytisine, and certain antidepressants, with bupropion showing the highest efficacy in supporting smoking cessation [174,175].

## 8. Selected Novel Treatment Possibilities

### 8.1. Canakinumab

Canakinumab is a monoclonal antibody that neutralizes interleukin 1 beta (IL-1β) signalling, thereby inhibiting the inflammatory pathway. It achieves this by directly targeting IL-1β. Additionally, canakinumab indirectly reduces the activity of matrix metalloproteinases (MMPs), vascular cell adhesion molecule (VCAM), intercellular adhesion molecule (ICAM), interleukin 6 (IL-6), and fibrinogen [176,177]. IL-1β plays a crucial role in intercellular communication within the immune system. It is a significant contributor to the immune response involved in atherosclerosis. In the vessel wall, IL-1β triggers the expression of adhesion molecules, cytokines, and chemokines, thereby amplifying the inflammatory response. Research indicates that IL-1β has a proatherogenic effect on vascular smooth muscle by inducing proinflammatory factors. Its systemic impact is primarily through inflammation driven by proinflammatory IL-6. The Canakinumab Anti-inflammatory Thrombosis Outcome Study (CANTOS) clinical trial investigated the potential of inhibiting IL-1β-induced inflammation for secondary prevention in patients at high risk of atherosclerotic ischemic events. The findings from these studies indicated a 15% reduction in the incidence of fatal stroke, non-fatal myocardial infarction, and cardiovascular mortality in patients administered canakinumab at a dose of 150 mg [178,179].

### 8.2. Propionic Acid

Another promising achievement was the isolation of the propionic acid metabolite from the intestinal microbiota, and then, using mouse models, observations were conducted. It was shown that propionic acid reduced cholesterol absorption, among others, in the intestines, and also influenced the increase in the population of regulatory T lymphocytes and the level of IL-10, which resulted in the inhibition of the main cholesterol transporter (Npc1l1) in the intestines, and consequently a reduction in total cholesterol and LDL cholesterol [1]. A randomised, double-blind, placebo-controlled study was also conducted in humans, which confirmed the beneficial effects [180].

### 8.3. Ziltivekimab

The study (RESCUE) examined the effect of ziltivekimab, a monoclonal antibody directed against IL-6 on the lymphocyte:neutrophil ratio (NLR), which is an indicator of inflammation. It was shown that ziltivekimab can inhibit IL-6, which may suggest a beneficial effect on inflammation associated with atherosclerosis [181].

### 8.4. Inclisiran

Inclisiran represents a pioneering small interfering RNA (siRNA) therapy aimed at selectively targeting and suppressing PCSK9 messenger RNA (mRNA) in the liver. This therapy works by specifically binding to N-acetylgalactosamine (GalNAc) and the asialoglycoprotein receptor (ASGPR) [182]. By inhibiting the expression of PCSK9, inclisiran enhances the production of hepatic LDL receptors, which boosts the removal of LDL cholesterol particles from the blood. This distinctive mechanism provides a promising strategy for lowering LDL cholesterol levels and decreasing cardiovascular risk in patients with ASCVD [183]. Studies have demonstrated that the use of inclisiran in patients with FH or ASCVD effectively reduces plasma LDL-C levels. This suggests that inclisiran therapy may lead to a decreased risk of cardiovascular events, including myocardial infarction (MI) [184].

### 8.5. Janus Kinase Inhibitors

The Janus kinase (JAK) family, consisting of JAK1, JAK2, JAK3, and Tyk2, comprises non-receptor protein-tyrosine kinases that facilitate signal transduction from cell membrane receptors to the nucleus through the JAK–STAT pathway. This signalling cascade plays a crucial role in regulating cell proliferation, differentiation, and apoptosis [185]. In smooth muscle cells (SMCs) within blood vessels, the JAK–STAT pathway contributes to proliferation in response to injury and angiotensin II-induced intracellular signalling. This indicates that JAK is significantly involved in the development of various vascular diseases, including systemic hypertension, post-angioplasty restenosis, and atherosclerosis [186]. Rodent studies have demonstrated that inhibiting JAK3 activity or expression decreases smooth muscle cell proliferation triggered by platelet-derived growth factor BB (PDGF-BB) and prevents injury-induced intimal hyperplasia [185]. The crucial role of JAK in vascular remodelling indicates that JAK inhibitors could potentially be used as treatments to prevent atherosclerosis and other vascular diseases in the future. Nonetheless, additional research is currently needed in this area.

## 9. Conclusions

The most common cause of mortality worldwide is undoubtedly CVD, which claims over 4 million lives annually in Europe alone. Among all components of CVD, MI, particularly type 1, is the most lethal.

Scientific evidence clearly indicates a link between chronic diseases such as CKD, hypertension, diabetes, and dyslipidemia, and lifestyle factors such as tobacco smoking, improper diet, and low physical activity leading to obesity. A better understanding of risk factors allows for the implementation of preventive measures, such as, among others, increasing physical activity, smoking cessation, or even taking selected medications to limit the development of atherosclerosis, thereby reducing the occurrence of serious complications such as MI.

Screening for CVD risk is important, and a systemic global CVD risk assessment is recommended in individuals with any major vascular risk factor. Different risk assessment models are used in different groups of patients. In apparently healthy people, the SCORE2 model is used for individuals below the age of 70 and SCORE2-OP is used for older people. For assessing the CVD risk in patients with an established ASCVD, it is recommended to use the SMART2 risk model.

The pathogenesis of type 1 heart attack is closely related to the destabilization of the atherosclerotic plaque, which is damaged, most often by rupture or erosion. The most typical symptoms of a heart attack are chest pain radiating from the left shoulder to the neck, shortness of breath, anxiety, heavy sweating, nausea, vomiting, or a combination of these symptoms. If the above symptoms occur, the patient should undergo a 12-lead ECG and the concentration of hs-cTn measured. In patients with acute coronary syndrome, we can use oxygen, sublingual nitrates and analgesics, and a loading dose of aspirin should be administered as soon as possible. It is very important to transport the patient to a centre performing PCI/CABG procedures. Complications of acute MI include cardiogenic shock, heart failure, re-infarction, and mechanical complications. 

## Figures and Tables

**Table 1:** Recommendations for CVD risk assessment by the ESC [33].

Recommendations	Class of Recommendation	Level of Evidence
Systematic global CVD risk assessment is recommended in individuals with any major vascular risk factor.	I	C
In the general population of men over the age of 40 and women over the age of 50 or postmenopausal without any known ASCVD risk factors, systematic or opportunistic CV risk assessment may be considered.	IIb	C
In patients who have undergone opportunistic CVD risk assessment, repeated screening after 5 years may be considered.	IIb	C
In adult individuals who are at risk of developing hypertension, an opportunistic screening of BP should be considered.	IIa	B
In the general population of men under the age of 40 and of women under the age of 50 without any known CV risk factors, systematic CVD risk assessment is not recommended.	III	C

Abbreviations: ASCVD—atherosclerotic cardiovascular disease; BP—blood pressure; CV—cardiovascular; CVD—cardiovascular disease. Classes of recommendation: Class I—evidence and/or general agreement that a given treatment or procedure is beneficial, useful, effective; Class IIa—weight of evidence/opinion is in favour of usefulness/efficacy; Class IIb—usefulness/efficacy is less well established by evidence/opinion; Class III—evidence or general agreement that the given treatment or procedure is not useful/effective, and in some cases may be harmful. Levels of evidence: B—data derived from a single randomised clinical trial or large non-randomised studies; C—consensus of opinion of experts and/or small studies, retrospective studies, registries.

**Table 2:** Patient categories and associated cardiovascular disease risk by the ESC [33].

Patient Category	Subgroups	Risk Categories
*Apparently healthy people*		
People without established ASCVD, diabetes mellitus, chronic kidney disease, familial hypercholesterolaemia	<50 years	Low to high risk
	50–69 years	Low to very high risk
	≥70 years	Low to very high risk
*Patients with chronic kidney disease*		
CKD without diabetes mellitus or ASCVD	Moderate CKD	High risk
	Severe CKD	Very high risk
*Familial hypercholesterolaemia*		
Associated with markedly elevated cholesterol levels	N/A	High risk
*Patients with type 2 diabetes mellitus*		
Patients with type 1 DM above the age of 40 may also be classified according to these criteria	Patients with well-controlled short-standing DM, no evidence of TOD, no additional ASCVD risk factors	Moderate risk
	Patients with DM without ASCVD and/or severe TOD, and not fulfilling the moderate criteria	High risk
	Patients with established ASCVD and/or severe TOD	Very high risk
*Patients with established ASCVD*		
Documented ASCVD, clinical or unequivocal on imagining	N/A	Very high risk

Abbreviations: ASCVD—atherosclerotic cardiovascular disease; CKD—chronic kidney disease; DM—diabetes mellitus; N/A—not applicable; TOD—target organ damage.

**Table 3:** Cardiovascular risk categories in apparently healthy people based on age according to SCORE2 and SCORE2-OP [33].

	<50 Years	50–69 Years	≥70 Years
**Low-to-moderate CVD risk**	<2.5%	<5	<7.5%
**High CVD risk**	2.5 to <7.5%	5 to <10	7.5 to <15%
**Very high CVD risk**	≥7.5%	≥10%	≥15%

Abbreviations: CVD—cardiovascular disease.

## Data Availability

The data used in this article were sourced from materials mentioned in the References section.
